# Effects of the EatWelLog^®^ Mobile Application for Managing Sarcopenic Obesity Among Community-Dwelling Older Adults: A Pilot Randomized Controlled Trial

**DOI:** 10.3390/nu18193243

**Published:** 2026-10-01

**Authors:** Justina Yat Wa Liu, Yixue Quan, Pui Ying Mak, Man Ki Law, Kenneth Ka Hei Lo, Tongyu Li, Jimmy Chun Yu Louie

**Affiliations:** 1 School of Nursing, The Hong Kong Polytechnic University, Kowloon, Hong Kong; yixue.quan@polyu.edu.hk (Y.Q.); pui-ying-bobo.mak@connect.polyu.hk (P.Y.M.); magky.law@polyu.edu.hk (M.K.L.); kenneth.kh.lo@polyu.edu.hk (K.K.H.L.); 2 School of Architecture and Design, Harbin Institute of Technology, Harbin 150006, China; litongyu@hit.edu.cn; 3 Department of Biomedical, Health and Exercise Sciences, School of Health Sciences, Swinburne University of Technology, Melbourne 3122, Australia; jimmylouie@swin.edu.au

**Keywords:** sarcopenia, obesity, exercise, diet, mHealth, intervention

## Abstract

**Background and Objectives**: Exercise combined with a hypocaloric high-protein diet is recommended for sarcopenic obesity (SO), yet accessible dietary support remains limited. EatWelLog^®^, a mobile health (mHealth) nutrition application, was developed to facilitate dietary self-management. This study evaluated the feasibility, acceptability, and preliminary effects of an EatWelLog^®^-supported lifestyle intervention for community-dwelling older adults with SO. **Methods**: In this two-arm randomized controlled trial (RCT), 40 adults aged ≥65 years at risk of SO were randomized to Conventional Supervised (CS) or mHealth-supported (MH) groups. Both groups received an 8-week intervention that included three individualized dietitian consultations, five group nutrition classes, and eight weekly 60 min multicomponent exercise sessions. The MH group also used EatWelLog^®^ during the intervention and the 6-month follow-up. Outcomes were assessed at baseline (T0), immediately post-intervention (T1), and at the 3-month (T2) and 6-month (T3) follow-ups. **Results**: Recruitment (48.8%) and attendance (83.3%) rates supported feasibility. Four participants in the MH group dropped out after the beginning of the intervention. The App demonstrated moderate-to-high acceptability. Between-group differences and group × time interactions were not detected for primary outcomes. Muscle strength improved from baseline to post-intervention in both groups, and physical performance showed improvements in MH across all follow-up assessments. Focus group participants valued the App’s dietary tracking, feedback, and reminders, though they identified limitations in food-recording functions. **Conclusions**: Findings support feasibility and acceptability, but strategies are needed to reduce attrition and improve compliance with recording diets. As this pilot was not powered for effectiveness, the App’s added benefit remains uncertain. A larger, adequately powered RCT is warranted.

## 1. Introduction

Sarcopenic obesity (SO), characterized by the coexistence of low muscle mass and excess adiposity, affects approximately 11% of older adults worldwide [1,2]. Compared with either sarcopenia or obesity alone, SO exerts a greater adverse impact on health, increasing the risk of falls, disability, knee and hip osteoarthritis, cardiometabolic diseases, institutionalization, and mortality [3,4]. SO is more prevalent in older adults, reaching 23% in individuals aged over 75 years [1]. This high prevalence makes older adults a particularly vulnerable group for whom effective management strategies are needed. Current evidence suggests that exercise and nutritional interventions are the primary lifestyle approaches for managing SO [5].

There is broad consensus that combining aerobic and resistance exercise is the most effective lifestyle strategy for managing SO [2,4]. Meta-analyses consistently demonstrate improvements in outcomes related to both sarcopenia and obesity [5,6,7]. Nevertheless, exercise alone is unlikely to optimize outcomes without appropriate nutritional support. Adequate protein intake supports muscle protein synthesis [8], while caloric restriction facilitates reductions in adiposity [9]. Developing an effective dietary strategy for SO presents a significant clinical challenge. Although hypocaloric diets have been shown to be effective in reducing adiposity [10,11,12], the resulting weight loss can exacerbate concomitant muscle wasting, thereby worsening the underlying sarcopenia [3,13]. Evidence indicates that pairing a hypocaloric diet with high-protein intake (i.e., 1.2–1.5 g/kg ideal body weight [IBW]/day) can prevent this loss of muscle mass, whereas a standard-protein (i.e., 0.8 g/kg IBW/day) hypocaloric diet often leads to further muscle depletion [10]. Nevertheless, while adequate protein preserves muscle quantity (mass), it is insufficient for improving muscle quality (strength) and overall physical function. For instance, evidence regarding the effects of a hypocaloric high-protein diet on muscle strength was mixed: one study reported an increase [11], while another observed no improvement from baseline [10]. Collectively, these findings suggest that dietary modification alone is unlikely to produce meaningful improvements across all SO-related outcomes. Consequently, combining aerobic and resistance exercise with a hypocaloric, high-protein diet is considered fundamental to contemporary SO management, as this multifaceted approach not only reduces fat mass and mitigates weight-loss-associated muscle wasting but also provides the essential mechanical stimulus needed to enhance muscle strength and physical performance [2,13,14].

Achieving meaningful clinical benefits from this multifaceted approach ultimately depends on sustained long-term changes in behavior. However, maintaining long-term adherence through conventional face-to-face dietary interventions remains challenging. Common barriers include logistical constraints, declining health status, participant attrition, and substantial demands on healthcare resources [15]. To overcome these barriers and foster long-term habit formation, mobile health (mHealth) nutrition applications have emerged as accessible, scalable, and cost-effective alternatives [16]. Through real-time dietary tracking, self-monitoring, and personalized educational content, these applications can provide ongoing support for sustained changes in dietary behavior [17,18]. Systematic reviews and meta-analyses have consistently shown that mHealth applications promote healthy dietary behaviors and improve a range of health outcomes (e.g., BMI and blood pressure) across various populations, including those with cardiovascular disease, diabetes mellitus, and obesity [19,20]. Despite growing evidence supporting nutrition-focused mHealth interventions, no application has been specifically developed for older adults with SO. Most existing mHealth-based interventions for overweight and obese adults focus primarily on weight management [21], whereas research on malnutrition and sarcopenia has paid less attention to adults who are overweight or obese [22]. This creates a potential gap for older adults with SO, whose diets require balancing energy restriction with adequate protein intake. This population presents unique dietary challenges that may benefit from a dedicated mHealth solution.

Therefore, we developed the EatWelLog^®^ (Patent number: HK30130380, Patents Registry, Intellectual Property Department, HKSAR), an mHealth mobile application specifically designed to improve dietary adherence to a hypocaloric high-protein diet. The App was designed specifically for older adults with SO and was based on the behavioral change framework proposed by Klasnja and Pratt for mobile health tools [16]. Key features of the EatWelLog App include: (1) monitoring dietary habits by recording meals and snacks from a predefined food list; (2) delivering personalized online coaching and reminders from a multidisciplinary healthcare team; (3) providing health information through videos, concise displays, and reminder prompts; and (4) incorporating gamified elements (i.e., SO-related educational videos and food-knowledge games) to communicate health and nutrition information related to SO.

The pilot trial aimed to evaluate the feasibility, acceptability, and preliminary effects of the EatWelLog^®^ App for older adults at risk of SO. This study employed a two-arm, 8-week randomized design. We hypothesized that: (1) the EatWelLog^®^ App would be feasible and acceptable for use by older adults at risk of SO to improve adherence to specialized dietary regimens; and (2) participants using the App would demonstrate greater improvements in SO-related outcomes (i.e., muscle strength, muscle quantity, physical performance, BMI, waist circumference, and body fat percentage), as well as nutritional status, nutritional self-efficacy, dietary quality, health-related quality of life, and adherence to diet and exercise regimens, compared with participants who did not use the App while engaging in the same lifestyle intervention.

## 2. Materials and Methods

This study was reported in accordance with the CONSORT extension for randomized pilot and feasibility trials (Table S1) [23].

### 2.1. Trial Design

This study was a prospective, assessor-blinded, two-arm, 8-week parallel-group pilot RCT, with follow-up assessments at 3 months (T2) and 6 months (T3) post-intervention. Participants were randomly assigned to one of the two interventions. Those allocated to the conventional supervised (CS) group received a standard lifestyle intervention consisting of three personalized dietitian consultations and five group nutrition classes delivered over 8 weeks, together with eight weekly 60 min multicomponent exercise sessions. Participants in the mHealth-supported (MH) group received the same supervised intervention and were additionally instructed to use the EatWelLog^®^ App for daily dietary self-monitoring and self-management and were encouraged to continue using the App independently for a further 6 months to assess long-term engagement. Details of the interventions are described below in Section 2.4.

This trial was conducted between April 2024 and November 2025. It has been registered at ClinicalTrials.gov (NCT06360432; submission date: 20 March 2024). Ethical approval was obtained from the Institutional Review Board of the Hong Kong Polytechnic University (HSEARS20241106003). Before the baseline assessment, all participants received a detailed explanation of the study procedures and provided written informed consent.

Because of budget restrictions and limited availability of dietitians, and the aim of enhancing peer support, the original design of eight personalized dietary consultations was replaced with three personalized consultations and five group-based nutrition classes before the implementation of the intervention.

### 2.2. Participants

Community-dwelling older adults were recruited from local community health centers if they satisfied all of the following criteria: (1) age 65 years or older; (2) living in the community; (3) presence of sarcopenia risk according to the 2019 Asian Working Group Consensus [24], defined as at least one of: low handgrip strength (<28 kg for men or <18 kg for women); reduced skeletal muscle mass index (<7 kg/m^2^ for men or <5.7 kg/m^2^ for women); impaired physical performance (Short Physical Performance Battery score < 9); (4) presence of obesity based on WHO recommendations for Asian populations [25], defined as at least one of: BMI ≥ 25 kg/m^2^, waist circumference ≥ 90 cm (men) or ≥80 cm (women); body fat percentage > 30 [26]; (5) having a smartphone with internet access; and (6) ability to communicate, read, and write Chinese.

Excluded were those with: (1) major hearing and vision impairments; (2) diseases affecting digestion or food consumption, including severe heart or lung diseases, chronic kidney disease, diabetes, gouty arthritis, anorexia nervosa, bulimia nervosa, and depression; (3) use of medications known to alter eating habits, digestion, or metabolism; (4) alcohol use disorder; and (5) implanted medical devices that could be affected by bioelectric impedance analysis.

### 2.3. Sample Size

The primary objective of a pilot study is to explore feasibility; therefore, a formal calculation of sample size is generally not required [27]. Hertzog suggested that a minimum of 10 participants per group would be sufficient for a pilot study with feasibility aims, and at least 20 per group would be adequate to demonstrate intervention efficacy [28]. Accordingly, a minimum of 40 older adults at risk of SO who met the eligibility criteria were recruited.

### 2.4. Intervention Arms

Both study arms received the same 8-week multicomponent lifestyle intervention comprising a dietary modification program and group-based exercise training. The interventions for both groups were conducted at a local university. To prevent cross-contamination, participants in the two arms were scheduled on separate days to ensure they did not interact with one another.

During the 8-week dietary program, all participants received three 1 h, face-to-face consultations with the same registered dietitian, followed by five group nutrition classes led by trained research assistants. The dietary regimen focused on achieving moderate energy restriction and optimized protein intake. Specifically, participants were encouraged to reduce energy intake by approximately 12% relative to estimated daily energy requirements and to achieve a protein intake of 1.5 g/kg of body weight per day. Body weight was calculated as follows: For participants with a BMI lower than 30, actual body weight was used. For obese participants with a BMI 30 or higher, adjusted body weight was used. Adjusted body weight was calculated as ideal body weight (IBW) + 0.4 × (actual weight − IBW). For men, IBW = 50 kg + 2.3 kg × (height × 39.37 − 60); for women; IBW = 45.5 kg + 2.3 kg × (height × 39.37 − 60). To optimize protein synthesis, participants were advised to distribute protein intake evenly across meals, targeting 25–30 g of protein per main meal, including about 2.5–2.8 g of leucine per meal [29].

In the exercise program, participants took part in 1 h weekly supervised group sessions for 8 weeks. Each group comprised approximately 10 participants. Each exercise session followed a standardized procedure: a 10 min warm-up, a 20 min resistance training session using elastic bands, 20 min of brisk walking, and a 10 min cool-down period. The number of repetitions in resistance training and walking intensity were tailored to participants’ physical capacities, with progression adjusted according to individual tolerance and safety. To encourage continued physical activity outside the supervised sessions, participants received a YouTube link and a pamphlet demonstrating the exercises. They were encouraged to continue exercising for at least 30 min at home, 5 days per week.

Only MH group participants were instructed to use the EatWelLog^®^ App for daily diet self-management, in addition to the above supervised dietary and exercise programs. The EatWelLog^®^ App was developed using Klasnja and Pratt’s mHealth framework [16] and established behavior change techniques [30]. Core functions include: (1) dietary self-monitoring using a food database that combines local food options developed by our team and the United States Department of Agriculture database [31], with voice and AI-driven image-assisted logging; (2) automated nutrient tracking and real-time feedback; (3) reminder prompts (encouraging App use) following prolonged inactivity by a trained research assistant (RA) via WhatsApp (version 2.25), with reminders delivered once every two weeks if participants did not use the App after the intervention period; (4) messaging functions supporting interaction with the research team, family members, and peers; and (5) educational and gamified content on healthy food options. Participants attended a training session before the intervention and received a comprehensive user guide for ongoing reference.

### 2.5. Measurements

Data were collected at four timepoints by trained RAs who were blinded to the group allocations: baseline (T0), immediately after the 8-week intervention (T1), 3 months post-intervention (T2), and 6 months post-intervention (T3). Table 1 gives detailed descriptions of all outcome measures.

#### 2.5.1. Baseline Characteristics

At baseline, sociodemographic characteristics were recorded, including age, gender, marital status, education level, and frequency of dining out per week.

#### 2.5.2. Feasibility

Feasibility outcomes were measured as recruitment, attendance, retention, adverse events, and adherence. Adherence was assessed using two self-reported scales on exercise and dietary regimens, and compliance with the dietitian’s recommended intakes of calories, protein, and fat. In addition, when participants turned on the EatWelLog^®^ App and used its features, the App automatically recorded usage log data, which were also treated as feasibility outcomes. A diet-recording compliance rate was calculated as diet-recording days divided by total days (56 supervised days and 217 self-management days). For example, the compliance rate of 22 days recorded during the supervised period equals 22/56 = 39.3%. 

#### 2.5.3. Acceptability

To explore user experiences with the EatWelLog^®^ App, MH group participants completed a self-developed survey on App acceptability and usability at T3. Semi-structured focus group interviews at T1 (4–6 participants per group) further explored the App’s usability. The final sample size was determined by data saturation, with recruitment concluding once no new themes or insights emerged. To ensure privacy and encourage open discussion, interviews were held in a private meeting room at a local university and moderated by an RA experienced in focus group facilitation.

#### 2.5.4. Preliminary Effects

Primary outcomes included parameters of sarcopenia and obesity: muscle strength, muscle quantity, physical performance, BMI, waist circumference, and body fat percentage. Secondary outcomes include nutritional status, nutritional self-efficacy, dietary quality, and health-related quality of life.

### 2.6. Randomization

Participants were randomly assigned to either the CS or MH group by an independent biostatistician using a computer-generated random number sequence (www.random.org, accessed on 26 June 2024). The biostatistician had no clinical involvement in the trial. To prevent selection bias, group assignments remained concealed until recruitment and baseline assessments were conducted.

### 2.7. Blinding

Because the interventions involved active participation (dietary counseling, exercise training, and use of the App), it was not possible to blind either the participants or the instructors/dietitians to the group allocations. However, the outcomes assessors were strictly blinded and remained unaware of the intervention assignments while conducting assessments at all timepoints (T0–T3). To reduce the risk of cross-contamination between groups, access to the EatWelLog^®^ App was limited to MH group participants using individual login credentials. Personalized dietary plans were supervised by qualified dietitians to prevent sharing between participants.

### 2.8. Statistical Methods

All statistical analyses were conducted in R 4.5.2. Absolute numbers and percentages of participants were used to present recruitment, attendance, and retention rates. Means and standard deviations (or medians and interquartile ranges) were used to present subjective exercise/dietary adherence, nutrient intakes, App usage, and App acceptability and usability. Wilcoxon signed-rank tests were conducted to assess food-record-based adherence from T1 to T3. Wilcoxon rank-sum tests were also performed to examine differences between the MH and CS groups in actual and suggested intakes. Both analyses were based on unimputed data.

Semi-structured focus group interviews were digitally audio-recorded and transcribed verbatim. NVivo 15 (Lumivero, Frankfurt am Main, Germany) was used to manage the transcripts and facilitate the identification of codes. Inductive content analysis was applied to synthesize themes and subthemes. A trained RA conducted the coding in accordance with the established content analysis guideline [43]. The research team discussed the coding and identified themes to reach a consensus on the final themes.

To analyze the preliminary effects, the intention-to-treat principle was followed. The Shapiro–Wilk test was used to examine the normality of continuous variables. Baseline characteristics between the groups were compared using independent t-tests for normally distributed continuous variables, Mann–Whitney U tests for non-normally distributed continuous variables, and λ^2^ tests for categorical variables. To estimate intervention effects over time, linear mixed-effects models were fitted using the *lmer()* function, with group (CS vs. MH), time (T0–T3), and their interaction (group × time) specified as fixed effects, and participant ID included as a random intercept. The protocol proposed that any baseline demographic variables that differed significantly between groups would be included as covariates, but no such differences were observed (Table 1). Nonetheless, age and gender were included as covariates given their known associations with SO. Missingness in the primary outcomes was 39% in the MH group and 0% in the CS group. Multiple imputation was applied using the *missRanger()* function, which employs chained random forests. Five imputed datasets were created and analyzed. A *p*-value of <0.05 was considered statistically significant.

## 3. Results

### 3.1. Participants

**Figure 1:**
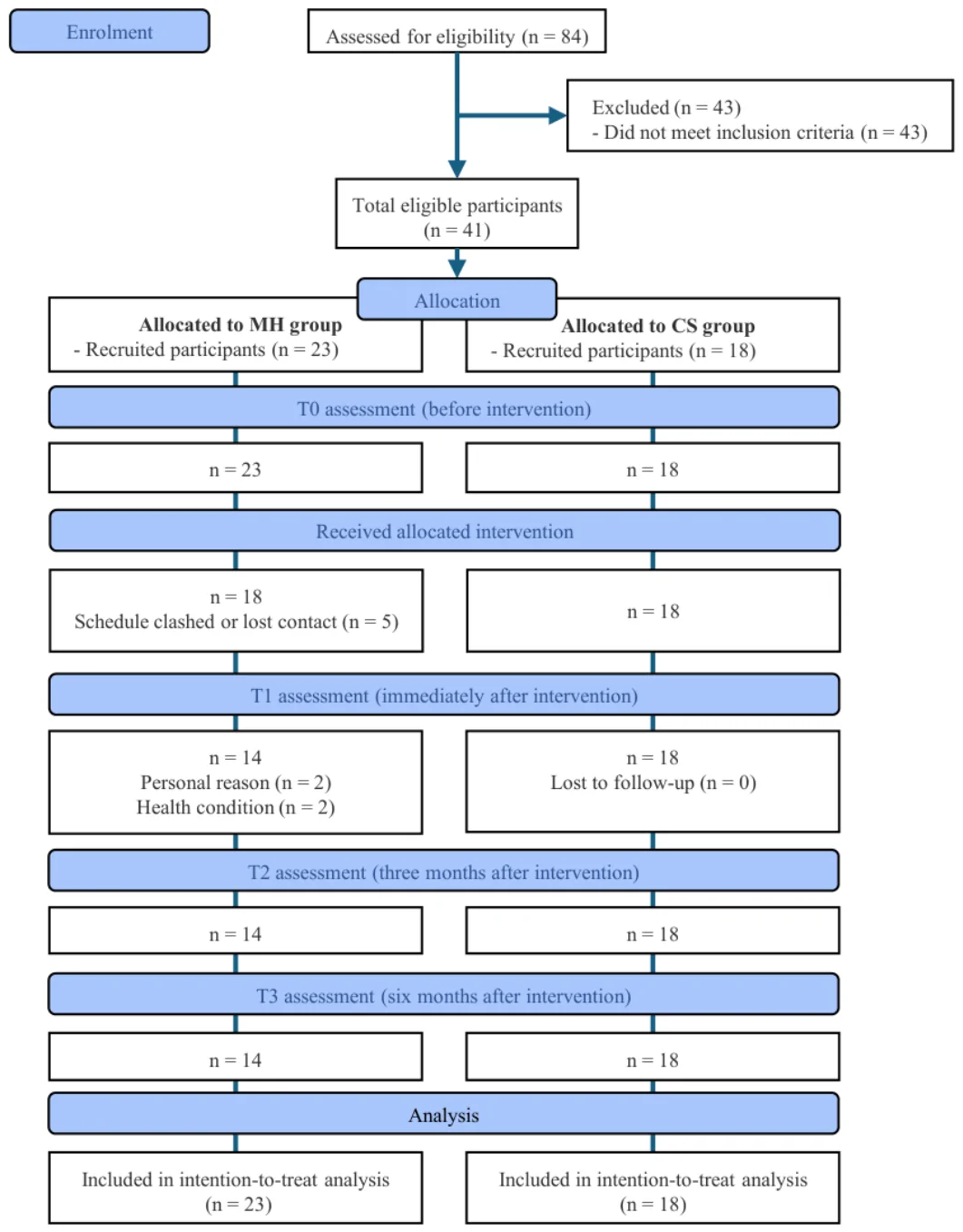
CONSORT flow diagram.

Figure 1 shows the CONSORT flow diagram. Forty-one eligible participants (33 females, 8 males) were enrolled in the trial. The mean age was 69.44 years (SD = 3.74). They were randomly assigned to either the MH or CS group. Twenty-three and 18 participants were enrolled in the MH and CS groups, respectively. Eighteen participants in the MH group received the intervention, while the remaining five dropped out at the beginning due to scheduling conflicts or loss of contact. Baseline demographic characteristics by group are summarized in Table 2. No significant between-group differences in demographic characteristics were found.

### 3.2. Recruitment

Screening and baseline assessments (T0) were conducted between April and July 2024, T1 assessments from August 2024 to May 2025, T2 assessments between November 2024 and August 2025, and T3 assessments between February and November 2025.

### 3.3. Feasibility

Recruitment lasted approximately 4 months, with a recruitment rate of 48.8% (41/84). The overall retention rate was 78% (32/41). In the MH group, five participants did not receive the allocated intervention from the beginning of the trial due to scheduling conflicts or loss of contact, and four participants attended few intervention sessions and missed all follow-up assessments because of personal reasons or health conditions unrelated to the trial. No significant differences were observed between dropout and retained participants in demographic characteristics or baseline primary outcomes (Table S3). All participants who completed the trial responded to all assessments. The proportion of participants attending at least six of the eight intervention sessions was 72.22% (13/18) in the MH group and 94.4% (17/18) in the CS group, with an overall attendance rate of 83.3% (30/36). No adverse events were reported by the participants throughout the study.

Self-reported exercise (M = 5.88, SD = 1.94) and dietary adherence (M = 6.9, SD = 1.45) remained moderate across the follow-ups, with no detectable group differences (Table 3). Food-record-based dietary adherence generally reflected lower energy intake and higher protein intake than prescribed in both groups (Table 4). Between-group differences were not detected (Table S4).

Descriptive statistics for App usage were summarized using median and interquartile range for Weeks 1 to 8 (when participants were enrolled in the supervised programs) and for Weeks 9 to 39 (when participants were no longer enrolled and were encouraged to use the App independently) in Table S5. App use was extended because participants continued until the final follow-up assessment; at one center, a typhoon delayed the 6-month assessment, prolonging usage to week 39 for some participants. During the supervised period, participants used the App for a median of 384 min and recorded meals on a median of 22 days across the 8 weeks, 77.8% (14/18) of participants watched the SO-related videos, and 61.1% (11/18) played the food-knowledge games. The compliance rates for recording diet were 40% and 8% during the supervised and self-management period, respectively. Average weekly use duration, diet-recording compliance rate, and the number of participants who watched the videos and played the games declined substantially once participants were no longer actively engaged in the supervised program.

### 3.4. Acceptability

Twelve out of 23 participants in the MH group completed the two self-developed scales on the acceptability and usability of the EatWelLog^®^ App. The scales ranged from one to five, with lower scores indicating greater acceptability or usability. The results showed that the mean acceptability was 1.96 (SD = 0.73) and the mean usability was 2.2 (SD = 0.38), indicating moderately acceptability and usability.

Two focus group involving ten MH group participants were conducted. Inductive thematic analysis revealed six themes and fifteen sub-themes (detailed with representative quotations in Table S6). The themes were: (1) achieve successful diet management using the EatWelLog^®^ App; (2) obstacles to food recording; (3) experiences with food-knowledge games; (4) experiences with videos on sarcopenic obesity; (5) views on adding a caregiver function; and (6) other comments about the App. A qualitative synthesis indicated that the App effectively improved diet management by enhancing awareness of the participants’ own dietary patterns through statistical presentations of current eating habits, motivating them to adopt healthier eating habits, and using the App as a reminder to manage meal arrangements and food choices to maintain healthy and appropriate eating habits. However, several systemic barriers were identified, such as limited food choices in the database, limited portion options to record amounts of intake, and a limited timeframe for recording food intakes. Regarding the features of food-knowledge games and SO-related educational videos, participants expressed a preference for face-to-face group-based modalities to sustain motivation for SO education. Most participants felt that it was unnecessary to add a caregiving function, regarding diet management as a personal responsibility and expressing concerns about access to their information. Overall, participants indicated that they would recommend the App to others, including those without SO, and expressed a desire to continue using it for diet management after the program.

### 3.5. Preliminary Effects

No significant between-group differences in primary or secondary outcomes were observed at baseline (see Table 3). Regarding sarcopenia criteria, 8 (19.5%) met the SPPB criterion, 39 (95.1%) met the handgrip strength criterion, and 6 (14.6%) met the SMI criterion. Overall, 30 participants (73.2%) met one criterion, 10 (24.4%) met two, and 1 (2.4%) met all three. For obesity, 27 participants (65.9%) met the BMI criterion, 34 (82.9%) met the waist circumference criterion, and 34 (82.9%) met the BF% criterion. In total, 9 participants (22%) met one obesity criterion, 10 (24.4%) met two, and 22 (53.7%) met all three.

Table 3 presents unadjusted scores for all primary and secondary outcomes across intervention groups from T0 to T3, together with results from the linear mixed-effect models. Outcome trajectories are shown in Figure S1. For primary outcomes, there were no significant group effects, while significant time effects were observed for waist circumference, muscle strength, and SPPB. Post-hoc pairwise comparisons were performed with Bonferroni corrections. Waist circumference significantly decreased from T0 to T1 (EMM = −2.92 [−5.53, −0.3], *t* = −2.18, *p* = 0.03), but then significantly increased from T1 to T2 (EMM = 3.78 [1.16, 6.39], *t* = 2.83, *p* = 0.005), and from T1 to T3 (EMM = 5.08 [2.47, 7.7], *t* = 3.81, *p* < 0.001) in the CS group. Muscle strength significantly increased from T0 to T1 in both the MH (EMM = 2.27 [0.26, 4.28], *t* = 2.24, *p* = 0.027) and CS groups (EMM = 2.76 [0.5, 4.87], *t* = 2.64, *p* = 0.008), and also increased from T0 to T2 (EMM = 2.13 [0.04, 4.22], *t* = 2.03, *p* = 0.046) and T0 to T3 (EMM = 2.68 [0.5, 4.87], *t* = 2.46, *p* = 0.017) in the MH group. SPPB significantly improved in the MH group from T0 to T1 (EMM = 1.15 [0.62, 1.68], *t* = 4.27, *p* < 0.001), from T0 to T2 (EMM = 0.91 [0.33, 1.5], *t* = 3.13, *p* = 0.003), and from T0 to T3 (EMM = 1 [0.78, 1.51], *t* = 3.83, *p* < 0.001). Among secondary outcomes, only EQ5D_VAS differed between groups, with higher scores in the CS group at T2 (EMM = 8.28 [0.57, 5.97], *t* = 2.13, *p* = 0.035).

## 4. Discussion

To our knowledge, EatWelLog^®^ is the first mHealth nutrition app specifically designed to support dietary self-management in older adults with SO. This two-arm pilot RCT found that the 8-week lifestyle intervention was feasible and acceptable among community-dwelling older adults at risk of SO. There were no significant group × time interactions for primary outcomes. Significant time effects were observed—muscle strength improved in both groups, and physical performance improved in the MH group. However, the pilot design does not allow firm conclusions on the App’s added effect. A fully powered RCT is therefore warranted.

### 4.1. Feasibility and Acceptability of the Intervention

The current recruitment and attendance rates support the feasibility of conducting a definitive trial. However, the attrition rate was notable in the MH group, which reduced the effective sample size and may have limited statistical power. Although baseline characteristics did not differ significantly between dropout and retained participants, selective attrition cannot be ruled out, and future trials should incorporate strategies to minimize dropout. To reduce participant heterogeneity, future trials should also adopt the updated AWGS 2025 criteria [44] and the Asia–Oceania consensus criteria for SO [45]. The significant declines in App compliance rate and weekly use duration during the self-management period suggest that long-term independent use was limited. Future trials should improve the App and incorporate strategies to increase sustained engagement, improve food-record completion, and reduce attrition.

Self-reported adherence was moderate throughout the follow-ups, while food-record-based adherence data suggested that participants generally achieved the prescribed dietary regimen of lower energy, higher protein, and lower fat intake [2,4]. However, many participants consumed considerably more protein than prescribed and maintained relatively poor DQI-I scores, suggesting that increased protein intake alone may not translate into improved overall diet quality. Regular dietary monitoring and feedback should therefore be incorporated into future trials to ensure safety and adherence.

Participants valued the App’s feedback, reminders, personalized goals, and self-monitoring functions. However, usability was limited by the restricted food database, inflexible logging options, small font sizes, and repetitive educational features. These findings provide clear targets for refinement before a definitive trial.

### 4.2. Preliminary Effects of the Intervention

Both groups showed improved muscle strength from baseline to immediately post-intervention. Similar short-term benefits of combined dietary and exercise interventions have been reported in systematic reviews or meta-analyses [46,47]. These findings support the potential value of integrating aerobic and resistance exercise with a hypocaloric, high-protein diet for improving muscle strength in older adults with SO. However, it is worth noting that muscle strength improvements were modest in absolute terms (MH group: improved from 15.3 to 18.5 kg; CS group: improved from 14.9 to 17.6 kg), with values remaining below or close to the sarcopenia threshold (<18 kg for women and <28 kg for men [24]).

Neither intervention significantly improved muscle quantity, as assessed by the skeletal muscle mass index. Improvements in muscle strength without detectable increases in muscle quantity may reflect early neuromuscular adaptations, whereas detectable changes in muscle quantity may require a longer intervention, a higher exercise dose, or a larger sample [6,46]. Physical performance improved from baseline to post-intervention in the MH group, whereas a statistically significant within-group improvement was not detected in the CS group. At the 3- and 6-month follow-ups, muscle strength and physical performance remained above baseline in the MH group. However, the corresponding between-group comparisons and group × time interactions were not statistically significant. These findings should be interpreted as preliminary within-group trends rather than evidence that the App caused or maintained the observed improvements. The small sample size further constrains the ability to determine whether these trends reflected a true intervention effect. A larger definitive trial is needed to estimate treatment effects with greater precision.

There were also no significant improvements in BMI, BF%, muscle quantity, nutritional status, nutritional self-efficacy, dietary quality, and health-related quality of life across both groups. Previous studies have shown that significant improvements in body composition generally require at least 150 min of exercise per week and more frequent dietary intervention sessions [48,49]. Similarly, although no dose–response analysis was performed, a 6-month RCT involving three group dietary sessions, one individual dietary session, and four exercise sessions per week demonstrated improvements in health-related quality of life and self-efficacy [50]. In the current 8-week trial, participants received three individual dietary consultations and eight 60 min exercise sessions. Thus, the frequency and length of the intervention were likely insufficient to produce meaningful changes in these outcomes.

Although self-reported exercise and dietary adherence did not differ significantly between groups, participants in the MH group tended to maintain fat intake closer to their individualized targets, whereas the CS group consumed significantly less fat than recommended during follow-up. This pattern paralleled the maintenance of muscle strength and physical performance in the MH group and raises the possibility that fat intake may play a role in sustaining muscle strength. A cross-sectional analysis of adults aged 20–59 reported an inverted U-shape association between fat intake and muscle strength [51]. However, because this finding was based on a different population and cannot establish causality, the potential link remains speculative and warrants investigation in future trials.

Two unexpected findings were noted. First, waist circumference decreased from baseline to immediate post-intervention in the CS group but not in the MH group. It then increased from immediate post-intervention to the 3- and 6-month follow-ups in the CS group, indicating that the short-term reduction during the active intervention period was not maintained. In contrast, the MH group showed no transient reduction despite continued dietary self-monitoring; instead, waist circumference increased about 2.44 cm at the 6-month follow-up. Thus, the two intervention groups followed different initial trajectories, although waist circumference ultimately exceeded baseline in both groups at the 6-month follow-up. The reasons for this asymmetrical response remain unclear, and dietary self-monitoring alone may not have been sufficient to reduce abdominal adiposity. The low intervention dose and relatively poor dietary adherence may again explain this result. That is, the intervention may have not been strong enough to counteract or delay the age-related increase in waist circumference [52].

Second, the CS group perceived better self-rated health than the MH group at the 3-month follow-up. The EQ5D_VAS scores declined from T1 to T2 and subsequently recovered at T3 in the MH group, whereas the scores in the CS group remained higher during follow-up than at baseline. Although participants in the MH improved in objective health outcomes (i.e., muscle strength and physical performance), they felt less positive about their health than those in the CS group after the intervention ended. In the CS group, participants only needed to follow the dietary and exercise program. In contrast, participants in the MH group also had to continue using the App after the active intervention phase. The technology issues reported in the focus group interview (e.g., limited time frame for recording food intake) may have increased the burden of App use. The App may also have increased participants’ awareness of their health and dietary behaviors [20], potentially leading to more critical self-assessments. These explanations remain speculative because the study did not directly examine the mechanisms underlying changes in perceived health. The isolated between-group difference may also reflect random variations resulting from the small sample size, the relatively wide confidence interval, and multiple outcome comparisons. Future trials should assess app burden, digital frustration, and perceived health longitudinally to clarify this finding.

### 4.3. Limitations

Several limitations should be considered when interpreting these findings. First, this was a pilot RCT with a small sample size, which may not have provided sufficient statistical power to detect between-group differences. A future definitive trial should incorporate a formal sample size calculation. Second, participants received relatively few sessions in both the individualized dietary consultations and multicomponent exercise sessions, and the intervention lasted only 8 weeks. This limited dose may explain some of the non-significant time effects. Although participants were encouraged to perform home-based exercise, this was not recorded or monitored by the research team, resulting in potential variability in adherence outside the one-hour weekly group exercise class. Future studies should increase both the frequency and duration of the intervention, systematically monitor participants’ exercise behaviors at home, and account for this potential influential factor when evaluating intervention effects. Third, in the MH group, an RA monitored the participants’ APP usage and sent WhatsApp reminders if they remained inactive. This additional contact during the follow-up period was not provided to the CS group, potentially introducing attention bias and making between-group differences partly attributable to this extra contact. Future trials should provide equivalent neutral contact to the CS group, such as standardized reminders to monitor food intake and physical exercise, to minimize attention bias. Fourth, participants reported several technological difficulties when using the App, such as a limited food database and rigid logging schedules. These issues may have discouraged use and should be improved in future revisions. Fifth, the broad SO eligibility criteria used in this study may have resulted in clinical heterogeneity. Participants were classified at risk of SO rather than having confirmed SO in this feasibility study. Future trials should adopt updated diagnostic criteria [44,45] and consider stratification by SO phenotype or severity. Sixth, comorbidity may influence outcomes such as SMI and physical performance [53], but it was not assessed, limiting the generalizability of the findings in older adults with multimorbidity. Future studies should include standardized comorbidity assessments to enhance generalizability. Seventh, the acceptability and usability scales used to evaluate experiences with the App were self-developed and have not undergone formal psychometric validation. Finally, participants were community-dwelling older adults who were willing and able to participate in a lifestyle and technology-supported intervention. The findings may not generalize to institutionalized adults, individuals with severe functional or cognitive impairments, or those with limited access to or experience with digital technology. The sample also showed a notable gender imbalance, with approximately 80% of participants being female, which may make the findings more representative of older women with SO.

### 4.4. Implications

This pilot study has several practical implications. First, combining individualized dietary counselling with group-based aerobic and resistance training can improve muscle strength in the short term. This supports further investigation of this approach in a larger-scale trial for managing SO in community-dwelling older adults. Second, the App may be considered as a potentially useful tool for self-monitoring and for supporting continued engagement with dietary behaviours. Third, the food-record-based adherence results showed a tendency toward over-restriction in calories. This highlights the need for careful monitoring of actual nutrient intake in older adults following weight-loss programs to ensure adequate energy levels.

## 5. Conclusions

This 8-week pilot RCT supports the feasibility and acceptability of a multicomponent lifestyle intervention incorporating the EatWelLog^®^ among older adults at risk of SO. The attrition rate (4/18) in the MH group was relatively high, and long-term independent App use was limited. No significant differences were observed between the MH and CS groups in the primary outcomes, but time effects were detected for improvements in muscle strength in both groups and physical performance in the MH group. Improvements in muscle strength and physical performance were maintained at 3 and 6 months in the MH group. Whether the EatWelLog^®^ provides additional benefits beyond the supervised intervention requires evaluation in a larger, adequately powered trial. These preliminary findings support further evaluation of the EatWelLog^®^ as a dietary self-management tool in a larger, adequately powered RCT. The future RCT examining the effectiveness of the EatWelLog^®^ should adopt the updated diagnostic criteria for SO, monitor at-home exercise behaviours, ensure equivalent neutral contact across groups, and implement strategies to reduce attrition and improve compliance with dietary recording.

## 6. Patent

EatWelLog^®^. Patents Registry, Intellectual Property Department, HKSAR (Patent number: HK30130380).

## Figures and Tables

**Table 1:** Summary of outcome measurements.

Outcome Domains	Outcome Measures	Measurement Descriptions
**Feasibility**	Recruitment	Length of recruitment and recruitment rate.
	Attendance	Attendance in the face-to-face intervention sessions.
	Retention	The rate of completion of follow-up assessments.
	Adverse events	Participants were encouraged to report any harmful events or adverse consequences during the trial.
	Subjective exercise adherence	Self-report on a 10-point Likert scale (0 = bad, 5 = moderate, 10 = good) for the item “the overall adherence to the exercise regimen”.
	Subjective dietary adherence	Self-report on a 10-point Likert scale (0 = bad, 5 = moderate, 10 = good) for the item “the overall adherence to the dietary regimen”.
	Food-record-based adherence	It was evaluated by comparing the individualized recommendations on nutrients established by the dietitians with the participants’ actual daily intakes, derived from three-day dietary records (see the DQI-I description below).
	App usage log data	The automatically recorded usage data included the daily frequency of using the App, weekly use duration (minutes), number of days with diet record entries per week, number of days watching SO-related educational videos per week, and number of days spent playing food-knowledge games per week.
**Acceptability**	Self-developed acceptability scale	The scale includes seven items: (1) I like this App; (2) I want to continue using this App; (3) This App helps me to eat healthily; (4) I like the daily diet report; (5) I like the daily statistics page; (6) I like the weekly statistics page; (7) I like the overall navigation of the screen. The items were rated on a five-point scale from 1 (strongly agree) to 5 (strongly disagree), with lower scores indicating greater acceptability.
	Self-developed usability scale	The scale includes eight items: (1) This app is easy to understand; (2) I feel comfortable when using this App; (3) I can take photos of my meals at home; (4) I can take photos of meals when eating out; (5) I can take photos of my meals when dining with family; (6) I can separate photos of the food I eat from the food others eat; (7) I trust the feedback this App provides about my diet; (8) I believe the App offers useful information on healthy eating. The items were rated on a five-point scale from 1 (strongly agree) to 5 (strongly disagree), with lower scores indicating greater usability.
	Semi-structured focus group interview	The semi-structured interview questions were developed by the research team based on the specific features of the EatWelLog^®^ App and the behavior change techniques underlying the intervention, with the aim of systematically identifying facilitators and barriers to dietary adherence. Table S2 contains a summary of the interview questions.
**Preliminary effects**		
***Primary outcomes***		
Muscle strength	Handgrip strength (kg)	It was measured using a TKK5401 dynamometer (Takei Scientific Instruments Co., Ltd., Tokyo, Japan). Participants performed three maximal handgrip trials with their dominant hand, each lasting approximately five seconds, with a rest interval of 10–20 s between trials. The average of the last two trials (kg) was used in the analyses.
Muscle quantity	Skeletal muscle mass index (SMI) (kg/m^2^)	SMI was calculated as appendicular skeletal muscle mass (ASM) divided by height squared (kg/m^2^) [32,33]. ASM was obtained using a multi-frequency bioelectric impedance analysis (InBody S10, InBody Co., Ltd., Seoul, Republic of Korea).
Physical performance	The Short Physical Performance Battery (SPPB)	The SPPB [34,35] was administered to assess lower limb function. It comprises the following components: standing for ten seconds with feet in three different positions, a 3 m walking speed test, and the time taken to rise from a chair for five times. Each component was scored and summed to yield a total score of 0 to 12, with higher scores indicating better physical performance.
BMI (kg/m^2^)		Body weight was measured to the nearest 0.1 kg using a calibrated Seca 770 scale, and height to the nearest 0.1 cm using a Seca 214 stadiometer (Seca GmbH & Co. KG, Hamburg, Germany). BMI was calculated as weight (kg) divided by height squared (m^2^).
Waist circumference (cm)		It was measured by measuring the midpoint between umbilicus and the xiphoid process, recorded to the nearest 0.5 cm.
BF%		It was obtained from a multi-frequency bioelectric impedance analysis using the Inbody S10.
***Secondary outcomes***		
Nutritional status	The Mini Nutritional Assessment (MNA)	The MNA has demonstrated acceptable reliability in Chinese populations to identify malnutrition or risk of malnutrition (α = 0.71) [36]. Scores range from 0 to 30, with higher scores indicating better nutritional status.
Nutritional self-efficacy	Health-Specific Self-efficacy Scale	Nutritional self-efficacy was assessed using the 5-item HAPA nutritional self-efficacy scale subscale of the Health-Specific Self-efficacy Scale. Each item is scored on a 4-point Likert scale, and total scores range from 4 to 20, with higher scores indicating greater self-efficacy. The Chinese version of the HAPA scale has shown excellent internal consistency (α = 0.92) [37,38].
Dietary quality	The Dietary Quality International-Index (DQI-I)	The DQI-I has been validated in China [39]. Participants completed three-day dietary records (two weekdays and one weekend day). Nutrient intakes were analyzed using the ESHA Food Processor software (version 11.16, Trustwell, OR, USA), and daily values were averaged across the three days. The DQI-I scores (0–100) were calculated from the average nutrient intakes, with a higher score indicating better overall dietary quality.
Health-related quality of life	The EuroQoL (EQ5D_index)	The EuroQoL comprises five dimensions: mobility, self-care, usual activities, pain/discomfort, and anxiety/depression [40]. Each dimension has five response categories: no problem, slight problems, moderate problems, severe problems, and unable to/extreme problems. Responses on the five dimensions were converted into a utility index (EQ5D_index) using Hong Kong population-based preference weights [41] via the *eq5d* function in R [42]. The index ranges from values below 0 (states considered worse than dead) to 1 (full health), with higher scores indicating higher health utility.
	Imaginable health status (EQ5D_VAS)	Participants rated their current health status on a 0–100-point visual analogue scale (EQ5D_VAS), where 0 represents the worst imaginable health and 100 represents the best imaginable health.

**Table 2:** Demographic characteristics by intervention groups, presented as means (standard deviations) for continuous variables and counts (percentages) for categorical variables.

	MH Group (N = 23)	CS Group (N = 18)	Results
Age	70.1 (4)	68.5 (3)	*W* = 249.5, *p* = 0.27
Gender			*λ*^2^ = 0, *p* = 1
Male	4 (17.4%)	4 (22.2%)	
Female	19 (82.6%)	14 (77.8%)	
Marital status			*p* = 0.9
Single	2 (8.7%)	3 (16.7%)	
Married	15 (65.2%)	12 (66.7%)	
Widowed	4 (17.4%)	2 (11.1%)	
Divorced	2 (8.7%)	1 (5.6%)	
Frequency of dining out per week			λ^2^ = 2.81, *p* = 0.42
0–1	4 (17.4%)	2 (11.1%)	
2–3	11 (47.8%)	12 (66.7%)	
4–5	5 (21.7%)	1 (5.6%)	
>6	3 (13%)	3 (16.7%)	
Education level			*p* = 0.91
Primary level	4 (17.4%)	2 (11.1%)	
Secondary level	14 (60.9%)	11 (61.1%)	
University level	5 (21.7%)	5 (27.8%)	

**Table 3:** Raw mean scores (standard deviations) for primary and secondary outcomes by intervention group and time, baseline comparisons, and results from linear mixed-effect models.

	Time	MH Group	CS Group	Baseline Comparisons	Linear Mixed-Effect Model Results
**Adherence**					
Subjective exercise adherence (Range = 0–10, ↑Score = ↑adherence)	T1	5.93 (1.49)	6.33 (1.61)	(no T0 assessment)	Group: F(1) = 0.94, *p* = 0.332, *η_p_*^2^ = 0.003
	T2	5.71 (1.33)	5.56 (2.41)		Time: F(2) = 0.19, *p* = 0.899, *η_p_*^2^ = 0.014
	T3	5.14 (2.28)	6.39 (2.12)		Interaction: F(2) = 0.8, *p* = 0.498, *η_p_*^2^ = 0.045
Subjective dietary adherence (Range = 0–10, ↑Score = ↑adherence)	T1	6.93 (1.21)	6.94 (1.39)	(no T0 assessment)	Group: F(1) = 0.09, *p* = 0.759, *η_p_*^2^ = 0
	T2	7.00 (1.30)	6.89 (1.53)		Time: F(2) = 0.1, *p* = 0.957, *η_p_*^2^ = 0.008
	T3	6.79 (1.53)	6.83 (1.79)		Interaction: F(2) = 0.05, *p* = 0.985, *η_p_*^2^ = 0.002
**Preliminary primary outcomes**					
BMI (kg/m^2^)	T0	26.68 (3.51)	25.64 (3.43)	t = 0.95, *p* = 0.35	Group: F(1) = 0.7, *p* = 0.4, *η_p_*^2^ = 0
	T1	26.21 (3.90)	25.46 (3.38)		Time: F(3) = 0.18, *p* = 0.906, *η_p_*^2^ = 0.022
	T2	26.21 (3.90)	25.54 (3.49)		Interaction: F(3) = 0.02, *p* = 0.996, *η_p_*^2^ = 0.002
	T3	26.17 (3.83)	25.51 (3.57)		
Waist circumference (cm)	T0	88.70 (7.81)	88.56 (10.62)	t = 0.05, *p* = 0.96	Group: F(1) = 0.05, *p* = 0.827, *η_p_*^2^ = 0
	T1	86.75 (8.68)	85.64 (11.31)		Time: F(3) = 4.28, *p* = 0.009, *η_p_*^2^ = 0.215
	T2	87.46 (8.39)	89.42 (11.69)		Interaction: F(3) = 0.64, *p* = 0.594, *η_p_*^2^ = 0.031
	T3	91.14 (8.65)	90.72 (9.87)		
Body fat percentage (%)	T0	36.38 (7.77)	35.59 (6.39)	t = 0.36, *p* = 0.72	Group: F(1) = 0.01, *p* = 0.923, *η_p_*^2^ = 0
	T1	34.85 (8.90)	35.37 (6.75)		Time: F(3) = 0.51, *p* = 0.676, *η_p_*^2^ = 0.022
	T2	35.64 (8.89)	36.18 (6.97)		Interaction: F(3) = 0.12, *p* = 0.947, *η_p_*^2^ = 0.004
	T3	36.40 (8.91)	35.99 (6.59)		
Skeletal muscle mass index (kg/m^2^, ↑ = ↑function)	T0	6.73 (0.90)	6.48 (1.05)	t = 0.82, *p* = 0.42	Group: F(1) = 2.03, *p* = 0.154, *η_p_*^2^ = 0.001
	T1	6.71 (0.84)	6.51 (1.06)		Time: F(3) = 0.7, *p* = 0.559, *η_p_*^2^ = 0.084
	T2	6.56 (0.83)	6.39 (1.09)		Interaction: F(3) = 0.35, *p* = 0.792, *η_p_*^2^ = 0.038
	T3	6.50 (0.82)	6.47 (1.07)		
Muscle strength (kg, ↑ = ↑function)	T0	15.30 (6.69)	14.88 (5.86)	W = 229.5, *p* = 0.56	Group: F(1) = 1.32, *p* = 0.251, *η_p_*^2^ = 0.003
	T1	18.54 (4.15)	17.64 (6.34)		Time: F(3) = 4.67, *p* = 0.004, *η_p_*^2^ = 0.074
	T2	17.51 (4.88)	16.47 (6.43)		Interaction: F(3) = 0.28, *p* = 0.842, *η_p_*^2^ = 0.003
	T3	18.46 (3.92)	16.84 (6.01)		
SPPB (Range = 0–12, ↑Score = ↑function)	T0	10.52 (1.88)	11.44 (1.25)	W = 147.5, *p* = 0.08	Group: F(1) = 1.42, *p* = 0.233, *η_p_*^2^ = 0
	T1	11.71 (0.73)	11.94 (0.24)		Time: F(3) = 6.7, *p* < 0.001, *η_p_*^2^ = 0.088
	T2	11.36 (1.34)	11.56 (0.86)		Interaction: F(3) = 1.63, *p* = 0.183, *η_p_*^2^ = 0.017
	T3	11.57 (1.34)	11.78 (0.73)		
**Preliminary secondary outcomes**					
HAPA (Range = 4–20, ↑Score = ↑self-efficacy)	T0	15.74 (3.32)	16.39 (2.50)	W = 187.5, *p* = 0.61	Group: F(1) = 1.13, *p* = 0.288, *η_p_*^2^ = 0
	T1	17.21 (1.93)	16.94 (3.70)		Time: F(3) = 1.36, *p* = 0.261, *η_p_*^2^ = 0.036
	T2	15.71 (3.47)	17.61 (2.50)		Interaction: F(3) = 1.12, *p* = 0.341, *η_p_*^2^ = 0.023
	T3	17.36 (2.37)	17.28 (2.44)		
MNA (Range = 0–30, ↑Score = ↑nutritional status)	T0	25.89 (2.14)	25.83 (1.85)	t = 0.09, *p* = 0.93	Group: F(1) = 2.69, *p* = 0.101, *η_p_*^2^ = 0.005
	T1	25.75 (2.62)	26.83 (1.29)		Time: F(3) = 0.48, *p* = 0.7, *η_p_*^2^ = 0.021
	T2	25.43 (2.52)	26.78 (2.05)		Interaction: F(3) = 0.73, *p* = 0.535, *η_p_*^2^ = 0.025
	T3	26.39 (2.80)	26.50 (2.22)		
DQI-I (Range = 0–100, ↑Score = ↑diet quality)	T0	46.19 (12.43)	46.00 (9.24)	t = 0.05, *p* = 0.96	Group: F(1) = 1.95, *p* = 0.163, *η_p_*^2^ = 0.001
	T1	42.50 (11.93)	46.56 (7.78)		Time: F(3) = 1.72, *p* = 0.164, *η_p_*^2^ = 0.025
	T2	48.71 (9.56)	48.94 (7.87)		Interaction: F(3) = 0.43, *p* = 0.735, *η_p_*^2^ = 0.004
	T3	49.14 (9.26)	47.44 (10.09)		
EQ5D_index (Range = 0–1, ↑Score = ↑health utility)	T0	0.90 (0.13)	0.89 (0.11)	W = 227.5, *p* = 0.59	Group: F(1) = 0.17, *p* = 0.679, *η_p_*^2^ = 0
	T1	0.93 (0.09)	0.91 (0.09)		Time: F(3) = 0.74, *p* = 0.531, *η_p_*^2^ = 0.01
	T2	0.87 (0.13)	0.90 (0.08)		Interaction: F(3) = 0.45, *p* = 0.714, *η_p_*^2^ = 0.004
	T3	0.94 (0.13)	0.90 (0.12)		
EQ5D_VAS (Range = 0–100, ↑Score = ↑imaginable health)	T0	71.74 (12.89)	77.72 (13.13)	t = −1.46, *p* = 0.15	Group: F(1) = 4.27, *p* = 0.039, *η_p_*^2^ = 0.001
	T1	77.07 (7.63)	80.00 (11.90)		Time: F(3) = 2.07, *p* = 0.108, *η_p_*^2^ = 0.052
	T2	73.86 (10.04)	82.67 (8.27)		Interaction: F(3) = 0.68, *p* = 0.566, *η_p_*^2^ = 0.013
	T3	77.86 (13.82)	81.44 (9.00)		

*Notes*. The two groups are the MH (mHealth) and CS (Conventional Supervised) groups. T0 adherence was not assessed because the intervention had not yet started. DQI-I = Dietary Quality International-Index; EQ5D = The EuroQoL Health-related Quality of Life Scale; HAPA = Health Action Process Approach Nutritional Self-efficacy Scale; MNA = Mini Nutritional Assessment Scale; SPPB = The Short Physical Performance Battery. ↑ indicates an increase.

**Table 4:** Median (interquartile range) for suggested and actual intakes at T1-T3 for calories, protein, and fat in the MH and CS groups.

		MH Group	Wilcoxon Signed Rank Tests	CS Group	Wilcoxon Signed Rank Tests
Calories	Suggested intake	1950 (1700, 1950)		1950 (1950, 1950)	
	T1 actual intake	1791 (1382, 2006)	V = 75, 95% CI = [−99.49, 465], *p* = 0.173	1693 (1558, 1966)	V = 160, 95% CI = [166.45, 443.08], *p* < 0.001
	T2 actual intake	1472 (1263, 1924)	V = 93, 95% CI = [127.38, 680.77], *p* = 0.009	1411 (1333, 1724)	V = 159, 95% CI = [334.93, 641.64], *p* = 0.001
	T3 actual intake	1295 (1228, 1868)	V = 92, 95% CI = [97.38, 707.32], *p* = 0.011	1437 (1169, 1713)	V = 171, 95% CI = [404.06, 743.65], *p* < 0.001
Protein	Suggested intake	70 (67, 73)		77 (70, 83)	
	T1 actual intake	101 (82, 122)	V = 5, 95% CI = [−51.8, −15.98], *p* = 0.001	93 (80, 118)	V = 12, 95% CI = [−35.32, −10.5], *p* = 0.001
	T2 actual intake	79 (67, 98)	V = 27, 95% CI = [−28.67, 3.77], *p* = 0.119	80 (70, 91)	V = 67, 95% CI = [−16.78, 6.66], *p* = 0.442
	T3 actual intake	83 (68, 91)	V = 17, 95% CI = [−26.05, −2.72], *p* = 0.025	79 (65, 96)	V = 79, 95% CI = [−16.91, 9.17], *p* = 0.799
Fat	Suggested intake	65 (57, 65)		65 (65, 65)	
	T1 actual intake	63 (57, 79)	V = 39, 95% CI = [−14.27, 10.8], *p* = 0.675	70 (58, 77)	V = 82, 95% CI = [−9.76, 8.47], *p* = 0.899
	T2 actual intake	59 (45, 72)	V = 70, 95% CI = [−7.02, 14.34], *p* = 0.296	53 (47, 66)	V = 133, 95% CI = [0.77, 17.92], *p* = 0.038
	T3 actual intake	52 (40, 68)	V = 71, 95% CI = [−4.93, 20.18], *p* = 0.268	56 (49, 65)	V = 148, 95% CI = [4.21, 19.63], *p* = 0.005

*Notes.* MH = mHealth; CS = Conventional Supervised. N = 14 in the MH group and N = 18 in the CS group across T1, T2, and T3 assessments.

## Data Availability

The original contributions presented in the study are included in the article/Supplementary Material, further inquiries can be directed to the corresponding author.

## Supplementary Materials

The following supporting information can be downloaded at: https://www.mdpi.com/article/10.3390/nu18193243/s1, Table S1. CONSORT 2010 checklist: extension to randomized pilot and feasibility trials. Table S2. Focus group interview semi-structured questions about the EatWelLog® App. Table S3. Demographic characteristics and baseline primary outcomes of dropouts versus retained participants. Table S4. Median (interquartile range) for the difference between suggested and actual intakes at T1–T3 for calories, protein, and fat in the MH and CS group. Table S5. Median (interquartile range) App usage log data and between-period comparison test results. Table S6. Content analysis of the semi-structured focus group interview. Figure S1. Mean scores of outcomes by intervention group and assessment timepoints.

## Abbreviations

The following abbreviations are used in this manuscript:

SO	Sarcopenic obesity
CS	Conventional supervised group
MH	MHealth-supported group
SPPB	Short Physical Performance Battery
IBW	Ideal body weight
BF%	Body fat percentage
DQI-I	Dietary Quality International-Index
EQ5D	The EuroQoL Health-related Quality of Life Scale
HAPA	Health Action Process Approach Nutritional Self-efficacy Scale
MNA	Mini Nutritional Assessment Scale
