# Supplementary material for: Effects of the EatWelLog® Mobile Application for Managing Sarcopenic Obesity Among Community-Dwelling Older Adults: A Pilot Randomized Controlled Trial
Source: Nutrients. 2026 Oct 1;18(19):3243. doi: 10.3390/nu18193243 (PMC13667122; doi:10.3390/nu18193243)
Supplement: Supplementary file 1 [file nutrients-18-03243-s001.zip › nutrients-4561208-supplementary.pdf]

Supplementary Materials

Table S1. CONSORT 2010 checklist: extension to randomized pilot and feasibility trials

**Section/topic and Item No.**	**Standard checklist item**	**Extension for pilot trials**	**Page No. where item is reported**
Title and abstract			
1a	Identification as a randomised trial in the title	Identification as a pilot or feasibility randomised trial in the title	1
1b	Structured summary of trial design, methods, results, and conclusions (for specific guidance see CONSORT for abstracts)	Structured summary of pilot trial design, methods, results, and conclusions (for specific guidance see CONSORT abstract extension for pilot trials)	1
Introduction			
Background and objectives:			
2a	Scientific background and explanation of rationale	Scientific background and explanation of rationale for future definitive trial, and reasons for randomised pilot trial	2-4
2b	Specific objectives or hypotheses	Specific objectives or research questions for pilot trial	4-5
Methods			
Trial design:			
3a	Description of trial design (such as parallel, factorial) including allocation ratio	Description of pilot trial design (such as parallel, factorial) including allocation ratio	5
3b	Important changes to methods after trial commencement (such as eligibility criteria), with reasons	Important changes to methods after pilot trial commencement (such as eligibility criteria), with reasons	6
Participants:			
4a	Eligibility criteria for participants		6
4b	Settings and locations where the data were collected		6
4c		How participants were identified and consented	6

Section/topic and Item No.	Standard checklist item	Extension for pilot trials	Page No. where item is reported
Interventions:			
5	The interventions for each group with sufficient details to allow replication, including how and when they were actually administered		7-8
Outcomes:			
6a	Completely defined prespecified primary and secondary outcome measures, including how and when they were assessed	Completely defined prespecified assessments or measurements to address each pilot trial objective specified in 2b, including how and when they were assessed	9-10, Table 1
6b	Any changes to trial outcomes after the trial commenced, with reasons	Any changes to pilot trial assessments or measurements after the pilot trial commenced, with reasons	/
6c		If applicable, prespecified criteria used to judge whether, or how, to proceed with future definitive trial	/
Sample size:			
7a	How sample size was determined	Rationale for numbers in the pilot trial	6
7b	When applicable, explanation of any interim analyses and stopping guidelines		/
Randomisation:			
Sequence generation:			
8a	Method used to generate the random allocation sequence		10
8b	Type of randomisation; details of any restriction (such as blocking and block size)	Type of randomisation(s); details of any restriction (such as blocking and block size)	10
Allocation concealment mechanism:			

Section/topic and Item No.	Standard checklist item	Extension for pilot trials	Page No. where item is reported
9	Mechanism used to implement the random allocation sequence (such as sequentially numbered containers), describing any steps taken to conceal the sequence until interventions were assigned		10
Implementation:			
10	Who generated the random allocation sequence, enrolled participants, and assigned participants to interventions		10
Blinding:			
11a	If done, who was blinded after assignment to interventions (eg, participants, care providers, those assessing outcomes) and how		10
11b	If relevant, description of the similarity of interventions		/
Analytical methods:			
12a	Statistical methods used to compare groups for primary and secondary outcomes	Methods used to address each pilot trial objective whether qualitative or quantitative	10-11
12b	Methods for additional analyses, such as subgroup analyses and adjusted analyses	Not applicable	/
Results			
Participant flow (a diagram is strongly recommended):			
13a	For each group, the numbers of participants who were randomly assigned, received intended treatment, and were analysed for the primary outcome	For each group, the numbers of participants who were approached and/or assessed for eligibility, randomly assigned, received intended treatment, and were assessed for each objective	12, Figure 1

Section/topic and Item No.	Standard checklist item	Extension for pilot trials	Page No. where item is reported
13b	For each group, losses and exclusions after randomisation, together with reasons		12, Figure 1
Recruitment:			
14a	Dates defining the periods of recruitment and follow-up		12
14b	Why the trial ended or was stopped	Why the pilot trial ended or was stopped	/
Baseline data:			
15	A table showing baseline demographic and clinical characteristics for each group		Tables 2&3
Numbers analysed:			
16	For each group, number of participants (denominator) included in each analysis and whether the analysis was by original assigned groups	For each objective, number of participants (denominator) included in each analysis. If relevant, these numbers should be by randomised group	Figure 1
Outcomes and estimation:			
17a	For each primary and secondary outcome, results for each group, and the estimated effect size and its precision (such as 95% confidence interval)	For each objective, results including expressions of uncertainty (such as 95% confidence interval) for any estimates. If relevant, these results should be by randomised group	12-15
17b	For binary outcomes, presentation of both absolute and relative effect sizes is recommended	Not applicable	/
Ancillary analyses:			
18	Results of any other analyses performed, including subgroup analyses and adjusted analyses, distinguishing prespecified from exploratory	Results of any other analyses performed that could be used to inform the future definitive trial	/

Section/topic and Item No.	Standard checklist item	Extension for pilot trials	Page No. where item is reported
Harms:			
19	All important harms or unintended effects in each group (for specific guidance see CONSORT for harms)		/
19a		If relevant, other important unintended consequences	/
Discussion			
Limitations:			
20	Trial limitations, addressing sources of potential bias, imprecision, and, if relevant, multiplicity of analyses	Pilot trial limitations, addressing sources of potential bias and remaining uncertainty about feasibility	21
Generalisability:			
21	Generalisability (external validity, applicability) of the trial findings	Generalisability (applicability) of pilot trial methods and findings to future definitive trial and other studies	15-22
Interpretation:			
22	Interpretation consistent with results, balancing benefits and harms, and considering other relevant evidence	Interpretation consistent with pilot trial objectives and findings, balancing potential benefits and harms, and considering other relevant evidence	15-20
22a		Implications for progression from pilot to future definitive trial, including any proposed amendments	21-22
Other information			
Registration:			
23	Registration number and name of trial registry	Registration number for pilot trial and name of trial registry	2
Protocol:			

Section/topic and Item No.	Standard checklist item	Extension for pilot trials	Page No. where item is reported
24	Where the full trial protocol can be accessed, if available	Where the pilot trial protocol can be accessed, if available	/
Funding:			
25	Sources of funding and other support (such as supply of drugs), role of funders		23
26		Ethical approval or approval by research review committee, confirmed with reference number	22

Table S2. Focus group interview semi-structured questions about the EatWelLog® App

1) To what extent did you find the EatWelLog® App helpful?
2) Did the App help you self-manage sarcopenic obesity? Did your self-management change after using it?
3) How did using the App affect your motivation to adopt healthy dietary behaviors?
4) What were the strengths and weaknesses of the App?
5) How would you describe your experience of using the App? Which features did you like most and least, and why?
6) Did the App meet your expectations regarding its content and usability? What motivated you to continue using it?
7) How could the App be improved?
8) Would you continue using the App after completing the study? Why or why not?
9) Would you recommend the App to others with sarcopenic obesity? Why or why not?
10) Would it be helpful for your family members to view your dietary records and nutritional information in the App? Why or why not?

Table S3. Demographic characteristics and baseline primary outcomes of dropouts versus retained participants

	retained (N = 32)	dropouts (N = 9)	results
T0_BF%	35.62 (7.28)	37.51 (6.71)	t = -0.73, p = 0.475
T0_BMI	25.97 (3.63)	27.13 (2.80)	t = -1.02, p = 0.32
T0_waist circumference	88.19 (9.34)	90.22 (8.13)	t = -0.64, p = 0.531
T0_SMI	6.55 (0.98)	6.89 (0.91)	t = -0.98, p = 0.346
T0_handgrip strength	15.05 (5.78)	15.36 (8.18)	W = 158.5, p = 0.659
T0_SPPB	11.03 (1.67)	10.56 (1.74)	W = 171, p = 0.349
Age	69.22 (3.49)	70.22 (4.68)	W = 131, p = 0.692
Gender			$\lambda^2 = 0$, p = 1
Male	6(18.8%)	2(22.2%)	
Female	26(81.2%)	7(77.8%)	
Marital status			$\lambda^2 = 1.2$, p = 0.753
Single	4(12.5%)	1(11.1%)	
Married	20(62.5%)	7(77.8%)	
Widowed	5(15.6%)	1(11.1%)	
Divorced	3(9.4%)	0(0%)	
Frequency of dining out per week			$\lambda^2 = 0.65$, p = 0.885
0-1	4(12.5%)	2(22.2%)	
2-3	18(56.2%)	5(55.6%)	
4-5	5(15.6%)	1(11.1%)	
>6	5(15.6%)	1(11.1%)	
Education level			$\lambda^2 = 1.35$, p = 0.51
Primary level	4(12.5%)	2(22.2%)	
Secondary level	19(59.4%)	6(66.7%)	
University level	9(28.1%)	1(11.1%)	

Notes. BF% = body fat percentage; SPPB = The Short Physical Performance Battery; SMI = skeletal muscle mass index.

Table S4. Median (interquartile range) for the difference between suggested and actual intakes at T1-T3 for calories, protein, and fat in the MH and CS group

Nutrients	Time	MH group	CS group	Wilcoxon rank sum tests
Calories	T1	-177 (-568, 149)	-306 (-493, -201)	W = 142, 95%CI = [-186.49, 457.17], p = 0.561
	T2	-478 (-687, -12)	-539 (-617, -277)	W = 132, 95%CI = [-237.1, 405.24], p = 0.837
	T3	-475 (-722, -65)	-656 (-781, -304)	W = 154, 95%CI = [-158.52, 517.92], p = 0.301
Protein	T1	30 (17, 52)	21 (5, 36)	W = 150, 95%CI = [-11.63, 29.46], p = 0.377
	T2	11 (-4, 33)	2 (-12, 19)	W = 149, 95%CI = [-12.8, 27.24], p = 0.398
	T3	17 (0, 21)	-1 (-14, 17)	W = 163, 95%CI = [-5.88, 30], p = 0.168
Fat	T1	-1 (-9, 16)	2 (-13, 10)	W = 129, 95%CI = [-12.62, 14.38], p = 0.925
	T2	-5 (-20, 0)	-12 (-18, -2)	W = 143, 95%CI = [-7.24, 15.21], p = 0.536
	T3	-9 (-24, 11)	-10 (-23, -1)	W = 142, 95%CI = [-10.94, 19.99], p = 0.561

Note. MH = mHealth; CS = Conventional Supervised. Values represent actual intakes subtracted by the suggested intakes. A lower value indicates that the actual intake was lower than the suggested intake, while a higher value indicates that the actual intake was higher than the suggested intake.

Table S5. Median (interquartile range) App usage log data and between-period comparison test results

	Weeks 1-8 (supervised period)	Weeks 9-39 (self-management period)	Between-period comparison tests
Total use duration (minutes)	384 (211, 460)	206 (27, 579)	W = 104, 95%CI = [-163.5, 191.2], p = 0.44
Average weekly use duration (minutes)	48 (26, 58)	7 (1, 19)	W = 171, 95%CI = [27.5, 53.5], $\boldsymbol{p}$ **< 0.001**
Number of days with diet records	22 (11, 34)	17 (1, 57)	W = 76.5, 95%CI = [-27, 9], p = 0.71
Diet-recording compliance rate (%)	40 (20, 62)	8 (0, 26)	W = 171, $\boldsymbol{p}$ **< 0.001**
Educational video watching (Y/N)	14/4	6/12	λ^2 = 5.51, $\boldsymbol{p}$ **= 0.019**
Food-knowledge game playing (Y/N)	11/7	2/16	λ^2 = 7.71, $\boldsymbol{p}$ **= 0.006**

Note. The App usage log data were analyzed based on 18 participants who received interventions in the MH group; the remaining five participants, who did not receive interventions, were excluded from this analysis.

Table S6. Content analysis of the semi-structured focus group interview

Themes and sub-themes	Quotations
1. **Achieve Successful Diet Management Using the EatWelLog® App**	
1a. Perceived helpfulness	"It is very helpful because it allows me to monitor myself. For example, if I want to lose weight, I can monitor my caloric intake, and the App helps me overcome difficulties. When I cannot find the exact food in the App, I select something similar to what I ate and try to make the record as accurate as possible. The App has some limitations, but I can work around them myself." (p004)<br>"This app can remind you to pay attention to your health. That is how I personally feel." (p007)<br>"In the past, I didn't know roughly how many calories were in different food. Now, when I eat a meal, I know how many calories it contains. Because of this App, it's much easier to make choices." (p006)
1b. Provide quantitative feedback	"It is good to have concrete data to look at. I don't know how accurate the data are, but having tangible data lets me know which nutrients I lack and which I consume excessively. It's clearer. In the past, I was simply told that I was a little overweight, but I didn't know what the problem was." (p002)<br>"That way, I know whether the portion I ate was appropriate, basically, whether I ate too much." (P006)<br>"You can see how much you have eaten, and adjust your intake at dinner accordingly." (p010)
1c. Motivation for diet management	**"It is helpful if you are willing to follow it properly. The problem is that not everyone wants to step out of their comfort zone. If you are willing to do so or make some sacrifice, it's not difficult to achieve your goals. it's all about personal effort."** (p004)<br>I really appreciate that the App provides us with customized goals. (p008)<br>"It is really good because I can specifically work on strengthening my muscles, which is very important at my age." (p006)
1d. Serving as a reminder	"This program can help people control their diet and their intake of salt, sugar, and other unhealthy foods, or good foods while encouraging healthier food choices. Often, you may talk about these things but then forget them. If you have a program that requires you to complete tasks daily or three to four times a week, it at least reminds you how to begin improving the quality of your diet." (p002)<br>"At least you will know how much healthy and unhealthy food you have actually eaten." (p007)
2. **Obstacles to Food Recording**	
2a. Limited food choices in the food database	"It would be ideal if the database contained more items so that I could directly enter a specific dish I ordered. Currently, some items can't be found because there are so many different foods, so we have to break the dishes into individual components." (p002)<br>"Sometimes the foods I eat aren't listed. I understand that it's difficult for the database to be completely precise, so sometimes you need to make adjustments yourself." (p010)

	"I often had to choose similar items instead, which might not accurately reflect what I had eaten." (p006)
2b Difficulty measuring food portions	Sometimes the items in the database aren't very detailed. For example, for some food items, I can't specify the exact portion, such as half a bowl of rice. I might eat one and a half or two bowls at a time." (p002)
2c. Limited timeframe for recording food intake	"I cannot go back and complete the record the next day. After midnight, you cannot return to the previous day's record." (p003)
	"The worst part is that, for example, if I complete only half of the record today and want to continue the next day, I can't return to the previous day to finish it." (p007)
	"Sometimes I forget to record my food intake. I want to complete the record later, but I just can't go back and fill it in." (p006)
**3. Experiences with Food-knowledge Games**	
3a. Games were too easy	"It seems there are only a few options and nothing new?" (p003)
	"It's too easy; there is no challenge." (p003)
	"I played it once out of curiosity, but I found it too simple to continue playing." (p004)
**4. Experiences with Videos on Sarcopenic Obesity**	
4a. Preference for face-to-face group sessions	"I prefer practical videos, such as exercise videos that teach you how to perform exercises or do simple tasks. However, I think doing the exercises together as a group would be more motivating. When you're at home watching the videos on your own, you tend to be lazier." (p004)
	"Honestly, I would only watch the videos without following the exercises. In group activities, everyone participates together, so you feel more engaged." (p004)
5. **Views on Adding a Caregiver Function**	
5a. Perceived as unnecessary	"For me, it would not make much difference." (p002)
	"I don't think monitoring is necessary. Those participating in this program probably do not need to be monitored." (p002)
	"If he (the husband) cooks for you, he already knows what you eat." (p010)
	"Actually, I don't mind third parties viewing information in the App, but I do have some doubts. For example, my domestic helper cooks at home. When I remind her, she remembers to use less sugar, salt, and oil. However, without reminders, she returns to her usual cooking practices" (P008)
5b. Privacy concerns	"There's no need. First, there are privacy concerns; second, I am responsible for my own health." (p004)
5c. Emphasis on personal responsibility	"Because I value my health. Another person might not have time to review my data, so self-monitoring is sufficient." (p002)
**6. Other comments about the App**	

6a. Suggestions for additional functions	"As my classmate just mentioned, could we have a 'My Preferences' section? It could store food items that I frequently record. I could then find them under 'My Preferences' instead of searching the entire database each time." (p002)
6b. Display and App Settings	"The font size in the survey and goal-setting sections is too small." (P009)
6c. Willingness to recommend the App to others	"If it became widely available, I would recommend it to others because it is really helpful." (p004)<br>"It does not have to be used only by people with sarcopenia; anyone who wants to manage their diet can use it." (p002)<br>"I hope this project succeeds because, if it becomes widely available, it could greatly benefit Hong Kong's healthcare system. Helping people become healthier would make a significant contribution to healthcare." (p004)<br>"Yes, I think the App might be more suitable for people who frequently eat out. For example, it includes dishes such as 'dry-fried beef chow fun.' However, for someone like me who rarely eats out, I feel that the database is insufficient. I have to enter each ingredient individually, but I do not have the patience to do that." (p008)
6d. Intention to continue using the App after the program	"I would continue using it." (p004, p010)

Figure S1. Mean scores of outcomes by intervention group and assessment timepoints

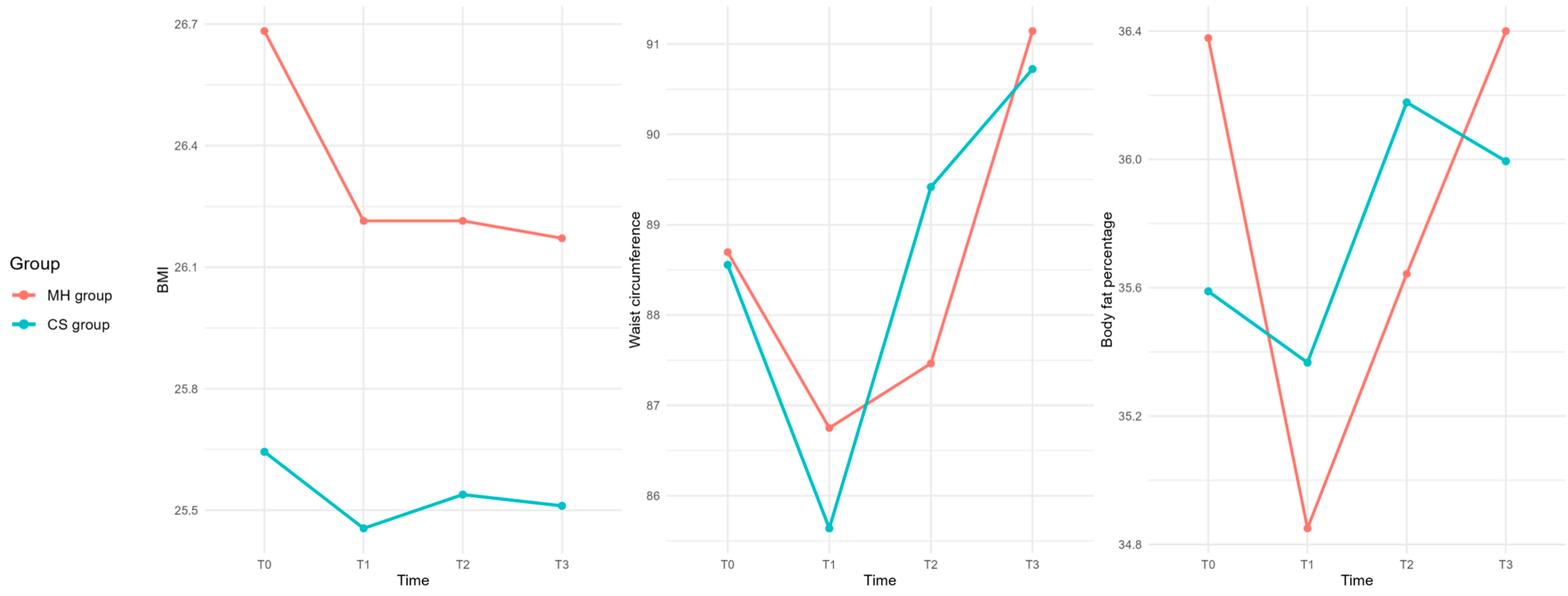

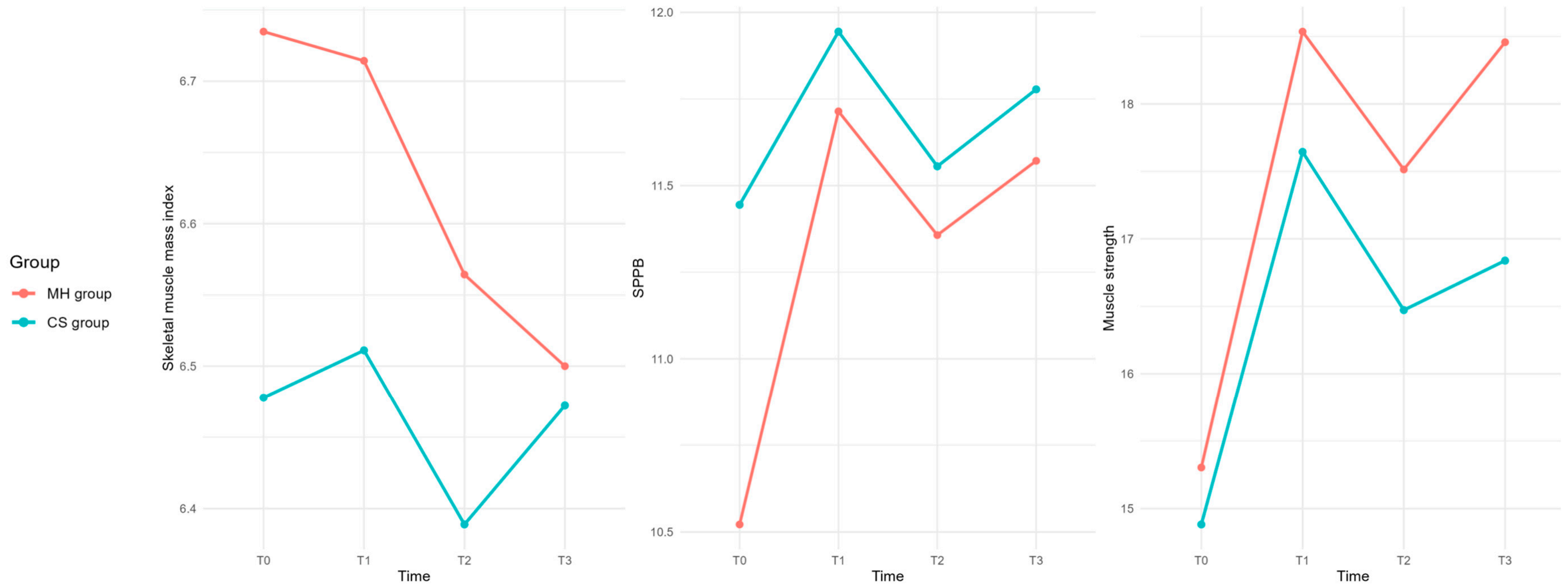

Group
MH group
CS group
Skeletal muscle mass index
6.7
6.6
6.5
6.4
T0
T1
T2
T3
Time
SPPB
12.0
11.5
11.0
10.5
T0
T1
T2
T3
Time
Muscle strength
18
17
16
15
T0
T1
T2
T3
Time

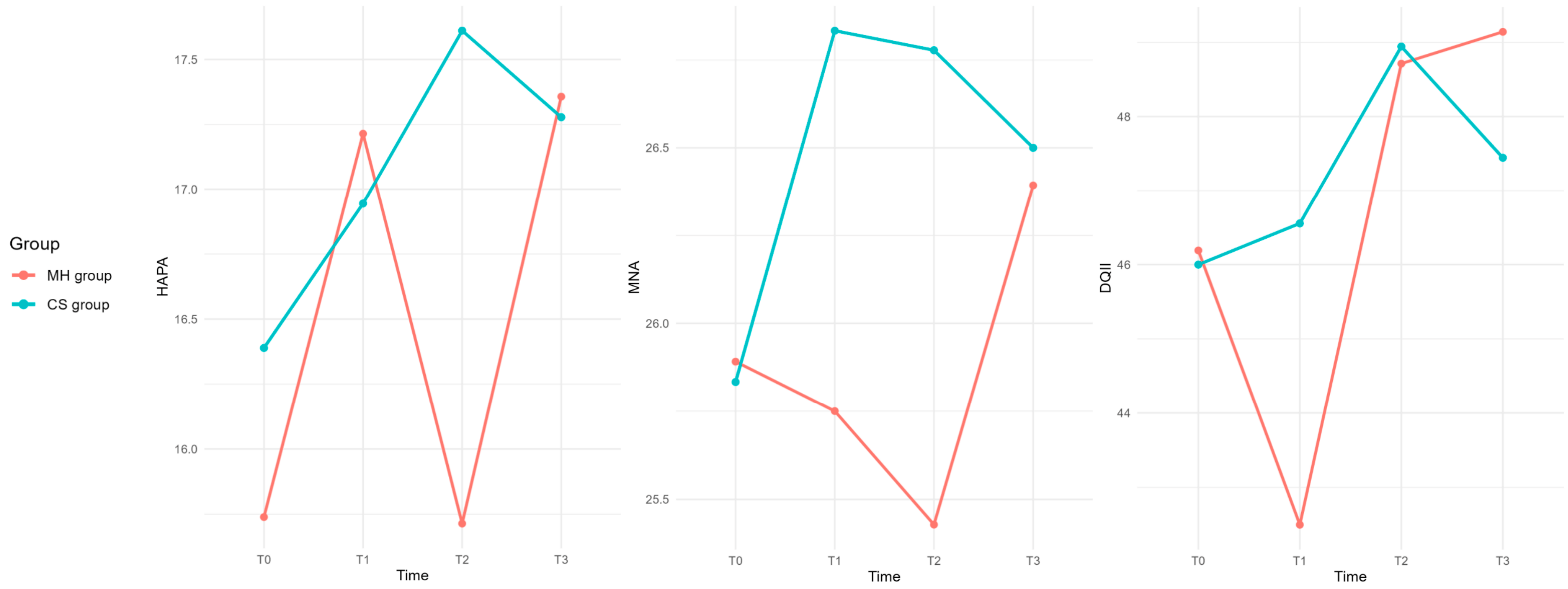

Group
MH group
CS group
HAPA
17.5
17.0
16.5
16.0
T0
T1
T2
T3
Time
MNA
26.5
26.0
25.5
T0
T1
T2
T3
Time
DQII
48
46
44
T0
T1
T2
T3
Time

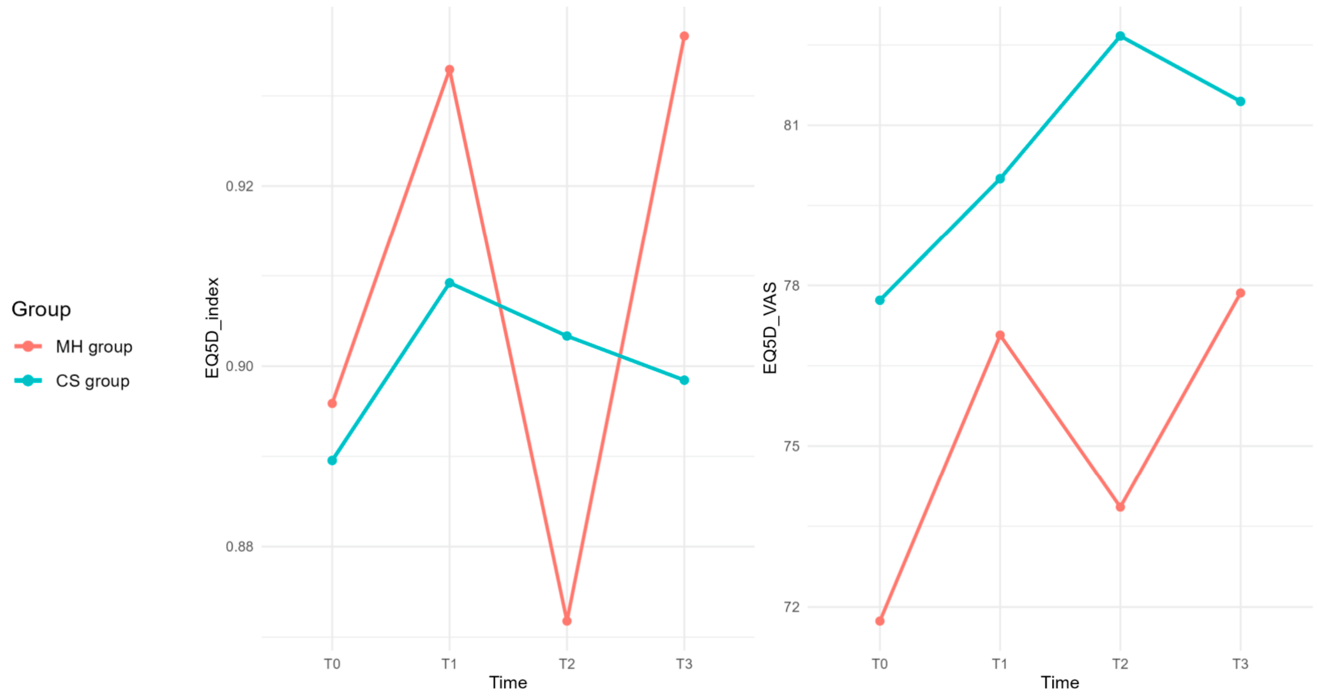

Notes. The two groups are MH (mHealth) and CS (Conventional Supervised) groups. DQI-I = Dietary Quality International-Index; EQ5D = The EuroQoL Health-related Quality of Life Scale; HAPA = Health Action Process Approach Nutritional Self-efficacy Scale; MNA = Mini Nutritional Assessment Scale; SPPB = The Short Physical Performance Battery.
